# Enhanced Transdermal Delivery of Lidocaine Hydrochloride via Dissolvable Microneedles (LH-DMNs) for Rapid Local Anesthesia

**DOI:** 10.3390/bios15080552

**Published:** 2025-08-21

**Authors:** Shengtai Bian, Jie Chen, Ran Chen, Shilun Feng, Zizhen Ming

**Affiliations:** 1 Microfluidics Research & Innovation Laboratory, School of Sport Science, Beijing Sport University, Beijing 100084, China; bst13@tsinghua.org.cn (S.B.); cr@bsu.edu.cn (R.C.); 2 Health Service Center of Qingshanhu Subdistrict Community, Hangzhou 311300, China; Chen_Jie2623@126.com; 3 Shanghai Institute of Immunology, Department of Immunology and Microbiology, Shanghai Jiao Tong University School of Medicine, Shanghai 200025, China; 4 State Key Laboratory of Transducer Technology, Shanghai Institute of Microsystem and Information Technology, Chinese Academy of Sciences, Shanghai 200050, China

**Keywords:** dissolvable microneedles, local anesthesia, analgesia, transdermal drug delivery, PVA

## Abstract

Microneedles represent an emerging transdermal drug delivery platform offering painless, minimally invasive penetration of the stratum corneum. This study addresses limitations of conventional lidocaine hydrochloride formulations, such as slow onset and poor patient compliance, by developing lidocaine hydrochloride-loaded dissolvable microneedles (LH-DMNs) for rapid local anesthesia. LH-DMNs were fabricated via centrifugal casting using polyvinyl alcohol (PVA) as the matrix material in polydimethylsiloxane (PDMS) negative molds, which imparts high mechanical strength to the microneedles. Biocompatibility assessments showed negligible skin irritation, resolving within 3 min. And drug-loading capacity reached 24.0 ± 2.84 mg per patch. Pharmacodynamic evaluation via mouse hot plate tests demonstrated significant analgesia, increasing paw withdrawal latency to 36.11 ± 1.62 s at 5 min post-application (*p* < 0.01). The results demonstrated that the LH-DMNs significantly elevated the pain threshold in mice within 5 min, surpassing the efficacy of conventional anesthetic gels and providing a rapid and effective solution for pain relief. These findings validate the system’s rapid drug release and efficacy, positioning dissolvable microneedles as a clinically viable alternative for enhanced transdermal anesthesia.

## 1. Introduction

Relentless progress in medical technology has elevated healthcare quality to paramount priority, positioning pain-free treatment as a transformative paradigm in modern clinical practice [1,2]. Local anesthetics are indispensable across diverse clinical scenarios—from minor surgical procedures and dermatological interventions to chronic pain management [3,4,5]. However, numerous routine clinical procedures, including skin biopsies [6], curettage [7], and aesthetic interventions, such as injectables [8] and laser therapies [9], often induce pain and discomfort, exacerbating patient anxiety and reducing treatment compliance. An optimal local anesthetic should possess the characteristics of rapid onset, long duration, ease of administration, and minimal side effects [10]. Lidocaine hydrochloride (LH) is one of the most widely used local anesthetics [11,12], offering low allergenic potential, swift action initiation, and moderate duration of action [13].

Currently, subcutaneous injection remains the clinical gold standard for achieving rapid and reliable anesthesia [14]. It presents limitations, including procedural trauma, specialized administration requirements, and biohazard waste generation [15,16,17]. While topical formulations, such as creams, sprays, and gels [18,19,20,21], offer non-invasive alternatives, but face stratum corneum barrier limitations, leading to delayed onset and suboptimal drug permeation [22,23,24]. Microneedle technology emerges as a promising platform to bridge this critical gap.

Microneedles represent a novel transdermal drug delivery system, utilizing arrays of micro-projections to transport therapeutic agents across the stratum corneum into the deeper layers of the skin [25,26]. Microneedles are broadly classified into five types: solid, hollow, coated, dissolvable, and hydrogel-based [25]. In terms of their delivery function, solid microneedles create microchannels in the skin to facilitate the diffusion of subsequently applied drugs [27,28,29]. Coated microneedles carry the drug on their surface [30], allowing for deposition into the skin upon insertion, though accurate dosage can be a challenge [31]. Hollow microneedles deliver drugs through a central channel [32], but they pose manufacturing difficulties and a risk of clogging. Additionally, all three types can generate sharp medical waste, raising biohazard concerns. Hydrogel microneedles swell [33] upon insertion into the skin, releasing the drug [34] gradually, but often having slower delivery rates and requiring longer application times [35]. Dissolvable microneedles (DMNs) are fabricated from drug-loaded biodegradable polymers [36,37]. Upon insertion, the microneedles dissolve quickly, enabling efficient intradermal drug release [38,39], providing a rapid, minimally invasive delivery option. Furthermore, DMNs eliminate the need for sharp waste disposal and reduce the risk of infection from reuse [25,40].

Delivering LH through a DMN platform loaded with the drug presents an optimal approach for achieving local anesthesia on the skin surface. Prior studies established foundational techniques for lidocaine delivery via MNs. Phase separation methods recently inhibited lidocaine diffusion into backing layers, yet faced scalability constraints, as demonstrated by Huang et al., who achieved 77.5% needle loading through interfacial tension control, but required precise solvent systems [41]. Concurrently Zhao et al. developed photo-crosslinked gelatin-methacryloyl hydrogel microneedles with backing layer reservoirs, achieving ultrahigh payloads of 2.9 mg/patch in neuropathic pain models [42]. While these approaches addressed diffusion and capacity limitations, they introduced manufacturing complexity or delayed onset. Compared to recent dissolving MN systems for lidocaine delivery [43], our LH-DMNs exhibit ultra-rapid onset with a higher drug load, addressing a critical gap in MN platforms, which typically require >30 min for initial effect. And we have adopted a more simplified manufacturing method while avoiding hygroscopic polyvinyl pyrrolidone (PVP).

In this study, two polymer materials, polyvinyl alcohol (PVA) and polyvinylpyrrolidone (PVP), were evaluated as base materials for DMNs. PVA is a water-soluble, synthetic, biodegradable polymer recognized for its non-toxicity and biocompatibility [44,45]. It is commonly employed in creating PVA-based hydrogel dressings for wound treatment through various crosslinking methods [46]. PVP, an inert, non-toxic, and biocompatible polymer, also exhibits favorable thermal stability, pH stability, and biodegradability. Additionally, it can encapsulate both hydrophilic and lipophilic drugs [47] and is frequently utilized in transdermal drug delivery systems [48]. Due to their advantageous physical, chemical, and biocompatibility properties, these two polymers are widely applied in the medical and pharmaceutical fields. In this study, a comparison between PVA and PVP was conducted to identify the more suitable polymer for the backbone of DMNs.

In terms of manufacturing technology, this study utilized a simple and cost-effective two-step method to fabricate lidocaine hydrochloride-loaded dissolvable microneedles (LH-DMNs). This approach ensured complete drug encapsulation within the needle tips while reducing the application time of the microneedle patch on the skin, thus enhancing user convenience. Optimized spatial distribution of the LH-DMNs further improved drug utilization efficiency. Both in vitro tests and in vivo studies with SD rats demonstrated that the LH-DMNs achieved promising performance in terms of skin penetration, drug delivery, and local anesthetic analgesic effects.

## 2. Materials and Methods

### 2.1. Instruments and Materials

Mask aligner (ABM/6/350/NUV/DCCD/M, ABM, San Jose, CA, USA); low-speed centrifuge (SC-3610, Zhongjia Scientific Instruments Co., Ltd., Hefei, China); vacuum dryer (PC-3, Yueci Electronic Technology Co., Ltd., Shanghai, China); electric thermostatic drying oven (Yiheng Scientific Instruments Co., Ltd., Shanghai, China); digital heating magnetic stirrer (HS7, IKA, Staufen, Germany); electronic balance (YH-A 6002, Yingheng Electric, Ruian, China); ultrapure water system (ZYMICRO, Runda Environmental Technology Co., Ltd., Xian, China); air compressor pump (ED-0908, Aidelun, Frankfurt, Germany); ultrasonic cleaner (GD-HTD240, Guangdian Ultrasonic Equipment Co., Ltd., Shenzhen, China); optical microscope (Leica Microsystems, Shanghai, China); universal testing machine (MC221/HY-0580, Hengyi Precision Instruments Co., Ltd., Shanghai, China); field emission scanning electron microscope (ISM-7500F, JEOL, Akishima-shi, Japan); high-performance liquid chromatography system (LC-20AD, Shimadzu, Kyoto, Japan); digital hot plate (ZH-YLS-6BS, Zhenghua Biological Instruments Co., Ltd., Huaibei, China); paraffin microtome (REM-710, Yamato Kogaku Co., Ltd., Asaka-shi, Japan); clean bench (Pudong Optical Instrument Factory, Shanghai, China).

Polydimethylsiloxane (PDMS, Dow Corning, Barry, UK); polyvinyl alcohol (PVA2488), polyvinylpyrrolidone (PVPk30), anhydrous ethanol, SU-8 photoresist, and lidocaine hydrochloride, all of analytical grade (Macklin Biochemical Technology Co., Ltd., Shanghai, China); ultrapure water (obtained from an ultrapure water system); ultraviolet (UV)-curable adhesive (8500, Ergo, Zurich, Switzerland); acetonitrile (chromatographic grade, Macklin Biochemical Technology Co., Ltd., Shanghai, China); porcine skin (from a local slaughterhouse); lidocaine hydrochloride gel (Beijing Zizhu Pharmaceutical Co., Ltd., Beijing, China); adult SD rats (male, weighing 200–270 g), mice (female, weighing 18–25 g), physiological saline, and sodium pentobarbital, all of analytical grade (Macklin Biochemical Technology Co., Ltd., Shanghai, China).

### 2.2. Preparation of Blank Microneedle Patches and LH-DMNs

#### 2.2.1. Selection of Microneedle Matrix Materials

Considering the functionality and application of the microneedle arrays, it was necessary to evaluate the storage stability of the microneedles under environmental conditions. PVP and PVA microneedles were stored separately at room temperature (25 °C) and standard atmospheric pressure for one month. The morphology of the microneedles was observed to assess the stability of the matrix materials.

#### 2.2.2. Preparation of Blank and LH-Loaded Microneedle Patches

Firstly, the primary microneedle master mold was fabricated using SU-8 negative photoresist via standard photolithography. Standard photolithography was performed using a mask aligner, involving ultraviolet exposure, post-exposure bake, and development to create the inverse microneedle structures. Subsequently, 10:1 *w*/*w* of PDMS prepolymer and curing agent was thoroughly mixed, degassed, poured onto the SU-8 master mold, and further degassed under vacuum to cast the PDMS negative mold. The PDMS was then cured in an oven for 1–2 h. After curing, the PDMS negative mold, containing the microneedle projections, was carefully peeled off of the SU-8 master mold. The preparation of microneedle patches was carried out using a two-step method. In the first step, a 200 μL solution of PVP or PVA was filled into the PDMS negative mold. The mold was then placed in a low-speed centrifuge and centrifuged at 3500 rpm for 5 min. After centrifugation, the mold was removed, and any excess matrix solution that had been expelled from the needle cavities was scraped off. The mold was left to air-dry at room temperature for 12 h to allow the needle matrix to dry completely. In the second step, 80 μL of UV-curable adhesive was gently applied to the surface of the dried PDMS mold, which provided mechanical support during application and enabled clean detachment after partial needle dissolution, followed by UV irradiation for 1 min to form a backing layer. After curing, the backing was demolded, and the microneedle formation was examined. The PVP mixture was prepared by dissolving 30% (*w*/*v*) polyvinylpyrrolidone (PVPk30) in a 20% (*w*/*v*) ethanol solution in a beaker. The PVA mixture was prepared by dissolving 15% (*w*/*v*) polyvinyl alcohol (PVA2488) in ultrapure water in a beaker. Due to the high viscosity and dissolution characteristics of PVA2488, stirring at 3000 rpm and heating at 80 °C were necessary. Homogeneous PVA matrix solution was obtained, followed by 2 h of undisturbed standing at room temperature.

For LH-DMN patches, a mixture containing 35% (*w*/*w*) LH and 15% (*w*/*w*) PVA was dissolved in 50% (*w*/*w*) ultrapure water. The solution was heated on a magnetic heating plate at 80 °C for 20 min to accelerate dissolution, followed by swelling at room temperature for 1 h. The mixture was then centrifuged at 3500 rpm for 5 min in a low-speed centrifuge to remove any air bubbles, resulting in a homogeneous matrix solution. Next, 200 μL of the matrix solution was filled into the PDMS negative mold and centrifuged at 3500 rpm for 5 min to ensure proper molding. The mold was air-dried at room temperature for 12 h. UV-curable adhesive was applied solely onto the mold surface (not needles), then cured via 60 s UV exposure to form a non-dissolvable backing layer. The UV-cured adhesive formed a non-dissolvable backing layer. After 3 min of skin application, the partially dissolved microneedle shafts exhibited reduced mechanical strength, enabling manual peeling of the intact backing layer. The dissolved needle matrix remained embedded in the skin for continuous drug release.

### 2.3. In Vitro Testing of Blank Microneedle Patches and LH-DMNs

#### 2.3.1. Morphological Characterization of LH-DMNs

For morphological analysis, a field emission scanning electron microscope (SEM) was used to capture images of the LH-DMNs after they were coated with a gold sputtering layer.

#### 2.3.2. Mechanical Performance Testing

The mechanical properties of the 10 × 10 microneedle patches were evaluated using a universal testing machine to measure the fracture force of the microneedles under compression. The microneedle patch was positioned on the testing platform with the needle tips facing upward. The MC221 testing machine (MC221/HY-0580, Hengyi Precision Instruments Co., Ltd., Shanghai, China) and control software HYtestV7.0 were configured to compression–displacement mode, and an axial force was applied using a probe at a speed of 0.5 mm/s until a preset displacement of 800 μm was reached. The trigger force was set at 0.05 N, and the force–displacement curve of the microneedles was recorded. The software report was then exported for further analysis.

#### 2.3.3. HPLC Analysis of LH-DMNs

The high-performance liquid chromatography (HPLC) analysis was performed using a Shimadzu LC-20AD system equipped with an Ultimate XB-C18 column (4.6 mm × 100 mm, 3 μm). The mobile phase consisted of a 50:50 (*v*/*v*) mixture of phosphate buffer and acetonitrile with pH 8.0. The phosphate buffer was prepared by diluting 1.3 mL of 1 M sodium dihydrogen phosphate solution and 32.5 mL of 0.5 M disodium hydrogen phosphate solution to 1000 mL with water. The flow rate was set at 1.0 mL/min, the column temperature was maintained at 30 °C, and the detection wavelength was 230 nm. A 5 μL injection volume was used for each sample. For the reference solution, an accurately weighed amount of lidocaine hydrochloride reference standard was dissolved in water and diluted to various concentrations. The sample solution was prepared by cutting the LH-DMNs into small pieces and placing them in a 100 mL volumetric flask, followed by the addition of water and ultrasonic treatment for 30 min. After thorough mixing, the solution was filtered to obtain the final sample for analysis.

A 5 μL aliquot of each sample, blank, and reference solution was injected, and the corresponding chromatograms were recorded. The reference standard was accurately weighed, dissolved in water, and further diluted with the mobile phase to prepare a series of standard solutions at concentrations of 0.2, 0.4, 0.6, 0.8, and 1.0 mg/mL. The peak area (X) was measured, and a linear regression analysis was performed using the peak area (X) against the concentration (Y).

Three prepared LH-DMN patches were selected and labeled as 1, 2, and 3. The samples were crushed, diluted, adjusted to a final volume of 100 mL, and filtered. The absorbance peak areas were then determined under the specified chromatographic conditions.

#### 2.3.4. In Vitro Skin Penetration Test of LH-DMNs

The skin penetration ability of the LH-DMNs was evaluated using porcine cadaver skin with 10 × 10 arrays of conical needles, measuring 800 μm in height [49]. The porcine skin was thawed in a 37 °C water bath for over 30 min and air-dried at room temperature for 10 min. After carefully removing the subcutaneous fat, the skin was cut into 1 cm thick samples, measuring 3 × 3 cm, and stored in physiological saline. The skin samples were laid flat on a clean workbench, with the stratum corneum facing outward, and the surface moisture was blotted dry.

To clearly visualize the microchannels after DMNs penetrate the skin, methylene blue solution was applied to the DMNs’ surface prior to conducting the penetration test. The microneedles were then inserted into the porcine skin by applying thumb pressure until complete needle insertion, confirmed by backing layer–skin contact, and maintained for 3 min. This state is biomechanically analogous to the universal testing machine-measured full compression point (20–25N). After insertion, the porcine skin was immediately stained with a small amount of methylene blue solution, and the excess dye was rinsed off using isopropanol. Finally, photographs of the stained porcine skin were captured for further analysis.

### 2.4. In Vivo Testing of LH-DMNs

All studies received ethical approbation from ethics committees.

#### 2.4.1. Skin Penetration Test

To evaluate the in vivo skin penetration performance of LH-DMNs, rat skin samples were prepared [50,51]. The rats were anesthetized via injection of sodium pentobarbital (50 mg/kg), and the hair on their backs was removed using a depilatory cream. The LH-DMNs were applied to the skin on the rats’ backs until complete needle insertion was confirmed by backing layer–skin contact, with the state maintained for five minutes before removal. The rats were immediately euthanized, and the skin that had been penetrated by the microneedles was carefully excised. The excised skin tissue was fixed in 10% formalin. After fixation, the tissue was embedded in paraffin, sectioned, and stained with hematoxylin and eosin (HE). The stained sections were then examined under a microscope to evaluate the effects of the microneedles on the skin tissue.

#### 2.4.2. Skin Irritation Test in Rats

To assess the potential skin irritation caused by LH-DMNs in rats, five rats were randomly selected and their back hair was shaved. The LH-DMN patches were applied to the shaved areas and gently pressed into the skin for three minutes before being removed. The sites of insertion were closely observed prior to removal of the patches and subsequently, at intervals of 30 s, 1 min, 2 min, and 3 min post-removal, to monitor any signs of irritation.

#### 2.4.3. Analgesic Test (Hot Plate Method)

The hot plate test was performed as previously described [52,53]. When subjected to thermal stimulation on the paw, mice display a pain response characterized by paw licking. Female mice were first placed on a hot plate set at 55 ± 0.5 °C, and the latency from the initial contact of the paw with the plate to the onset of paw-licking behavior was recorded as an indicator of pain threshold (measured in seconds). To exclude animals that were excessively insensitive or overly sensitive to heat, mice with latency periods of ≥30 s or ≤5 s were excluded from the study. Each mouse underwent two tests with a 5-min interval between them.

After selecting suitable subjects, 27 female mice (weighing 18–25 g) were randomly chosen and marked. Baseline pain response times were recorded before treatment, after which the mice were randomly divided into three groups: control, lidocaine hydrochloride gel (LH-GEL), and LH-DMN groups. Control group: Saline was injected into the paw. LH-GEL group: A gel containing the same concentration of LH as in the LH-DMNs was applied topically to the paw. LH-DMN group: Microneedle patches loaded with LH were applied to the paw for 5 min and then removed.

Pain response times were recorded at 5 and 10 min post-administration for each group. If no paw-licking behavior was observed within 60 s, the mouse was immediately removed from the hot plate, and the response time was recorded as 60 s. The change in pain threshold was calculated using the following formula: percentage increase in pain threshold = (T2 − T1)/T1 × 100%, where T1 represents the baseline pain response time, and T2 represents the post-treatment response time. The homogeneity of variance was first assessed for the raw data. Paired tests were conducted for within-group comparisons, and paired *t*-tests were used for comparisons between groups.

### 2.5. Data Analysis

Experimental data were presented as mean ± standard deviation (x ± s). The homogeneity of variance was first assessed for the raw data. Paired tests were conducted for within-group comparisons, and paired *t*-tests were used for comparisons between groups. A *p*-value of less than 0.05 was considered statistically significant.

## 3. Results

### 3.1. Characteristics of Blank Microneedle Patches and LH-DMNs

**Figure 1:**
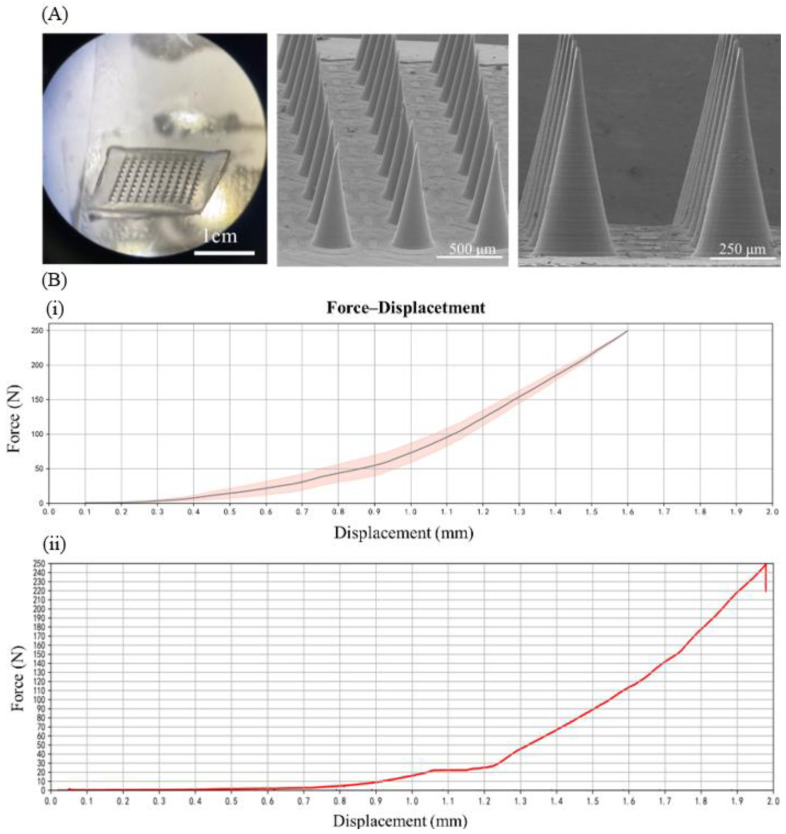
(**A**) Appearance of microneedle patch (left) and SEM images of LH-DMNs under different distance conditions; (**B**) force–displacement chart of LH-DMN patch, where (**i**) represents blank microneedles and (**ii**) represents LH-DMNs (*n* = 5).

The blank microneedle patch comprised a 10 × 10 array of conical needles, measuring 800 μm in height, 300 μm in base diameter, and ~5 μm in tip radius. Optical microscopy confirmed precise geometric uniformity, with intact needle tips and consistent dimensions, while post-drying structural analysis revealed that >95% of needles maintained intact morphology, with no visible fractures or tip defects under 40× optical microscopy, critical for reliable stratum corneum penetration (Figure 1A).

For viable clinical implementation, matrix materials must simultaneously fulfill three criteria: biocompatibility to ensure tissue safety, storage stability for shelf life viability, and rapid hydrosolubility for prompt drug release. Initial screening of PVP and PVA—both water-soluble polymers with established biocompatibility—uncovered a critical divergence: PVP microneedles underwent complete structural collapse within one month at ambient humidity due to hygroscopic degradation, whereas PVA counterparts maintained dimensional fidelity under identical conditions. This decisive stability advantage established PVA as the optimal matrix for subsequent drug-loaded fabrication.

### 3.2. Mechanical Performance Testing of Blank Microneedle Patches and LH-DMNs

Microneedles intended for transdermal drug delivery must possess sufficient mechanical strength to penetrate the skin without bending or breaking during the insertion process. The mechanical strength of the microneedles was assessed using a universal testing machine in compression mode, continuously recording the force–displacement data as compressive force was applied to the microneedle patches. The PVA microneedles demonstrated a mechanical strength of 52.25 ± 8.72 N (*n* = 5). Previous studies have shown that the force required to pierce the skin is approximately 0.045 N per needle [54]. The PVA microneedle patch, with a mechanical force of approximately 0.54 N failure force per needle, was more than sufficient for effective skin penetration. Additionally, the PVA microneedles exhibited gradual bending under compression without fracturing, indicating appreciable toughness (Figure 1B). After drug loading, changes in the matrix material resulted in altered mechanical strength, with the LH-DMNs showing a mechanical strength of 0.23 N failure force per needle (Figure 1B). Although the mechanical strength of the LH-DMNs was lower than that of the unloaded PVA microneedles, it remained adequate for successful skin penetration.

### 3.3. HPLC Analysis of LH-DMNs

**Figure 2:**
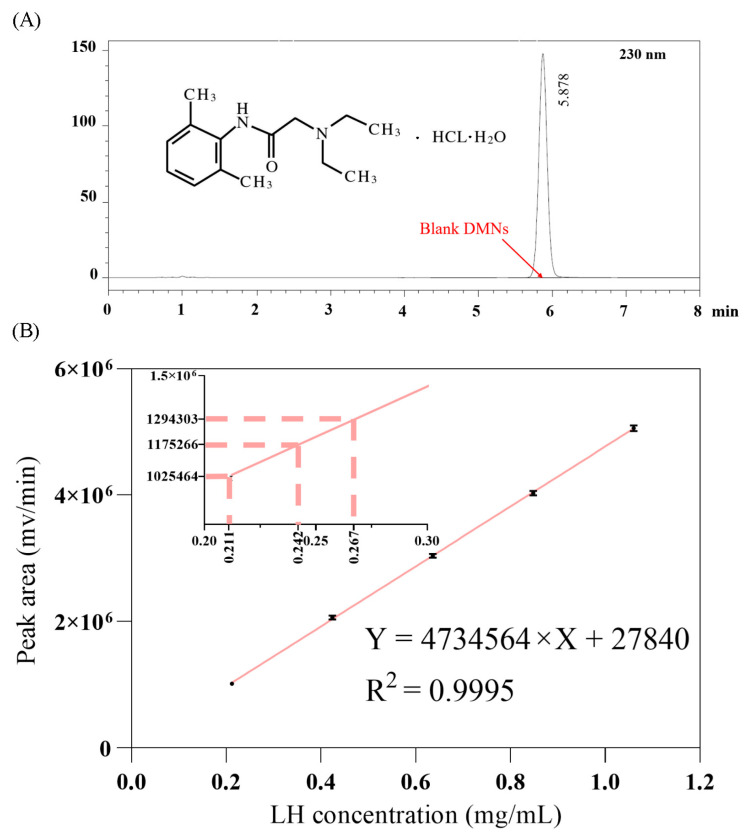
(**A**) Liquid chromatography results of blank solution and lidocaine hydrochloride and (**B**) the standard curve for lidocaine hydrochloride (*n* = 3); insert: drug loading in LH-DMNs; the axes correspond to the main figure.

In this study, high-performance liquid chromatography (HPLC) was employed to determine the lidocaine hydrochloride content in LH-DMNs. Under the chromatographic conditions described in Section 2.3.3, the results showed no interference from the excipients, with the lidocaine hydrochloride peak observed at 5.878 min (Figure 2A). This confirms that the chromatographic method is both accurate and reliable for measuring the drug content in the microneedle patches.

According to the standard curve experiment in Section 2.3.3, a concentration range of 0.2 to 1.1 mg/mL of LH standard solution was used. The data recorded in Figure 2B show that the solution concentration (X, in mg/mL) and the peak area (Y, in mV/min) exhibit a significant linear relationship. The regression equation is Y = 4,734,564 × X + 27840, with a correlation coefficient of R^2^ ≈ 0.99 (Figure 2B). This result indicates that lidocaine hydrochloride exhibited a good linear relationship within this concentration range, verifying the accuracy and reliability of this method for the quantitative analysis of LH.

Further analysis was conducted on the LH content in three LH-DMNs, labeled 1, 2, and 3. The experimental data, presented in Table 1, indicate that the average lidocaine hydrochloride content in each microneedle patch was 24.0 ± 2.84 mg (X ± SD). This drug content is theoretically sufficient to meet the requirements for local anesthesia and pain relief, indicating that the prepared microneedle patches have adequate drug loading to effectively achieve local anesthetic and analgesic effects [55,56].

HPLC was successfully used in this experiment to quickly and accurately determine the lidocaine hydrochloride content in LH-DMNs. The results showed consistent drug loading across the microneedle patches, indicating good stability and reproducibility in the manufacturing process. The high drug content of LH-DMNs suggests that they hold significant potential for local anesthesia and pain relief, providing a strong foundation for their further development and application as a painless, non-invasive local anesthetic delivery system.

### 3.4. Evaluation of LH-DMN Penetration

**Figure 3:**
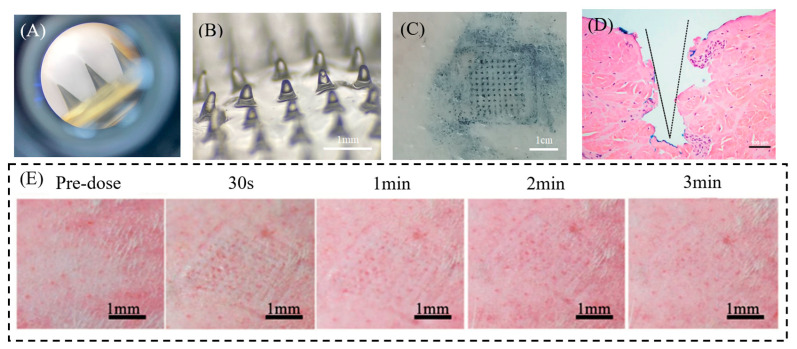
(**A**) Image before the application of LH-DMNs to porcine skin; (**B**) image after the application of LH-DMNs to porcine skin; (**C**) image of porcine skin penetration staining; (**D**) rat skin tissue section following microneedle insertion; (**E**) rat skin irritation test, showing the recovery of the skin over time after the application of LH-DMNs.

Figure 3A–C illustrates microneedle penetration dynamics and post-insertion morphology. The distinct indigo microchannels on porcine skin confirm successful stratum corneum perforation, demonstrating adequate mechanical strength. Critically, over 90% of LH-DMNs underwent complete dissolution within three minutes, enabling rapid drug liberation.

Histological analysis of SD rat skin revealed uniformly spaced conical pores corresponding to the microneedle array geometry, as shown in Figure 3D. These well-defined microchannels facilitate direct drug access to subcutaneous targets. Notably, skin elasticity caused tissue compression during insertion, reducing actual penetration depth to approximately 400 μm despite the 800 μm needle height. This elastic recoil phenomenon, while limiting absolute depth, did not compromise transdermal delivery efficiency.

Importantly, biocompatibility assessments documented only transient mild erythema at application sites, with no hemorrhage, infection, or persistent inflammation. All dermal reactions resolved spontaneously within three minutes, indicating favorable safety and rapid tissue recovery (Figure 3E).

### 3.5. In Vivo Anesthetic Evaluation of LH-DMNs

**Figure 4:**
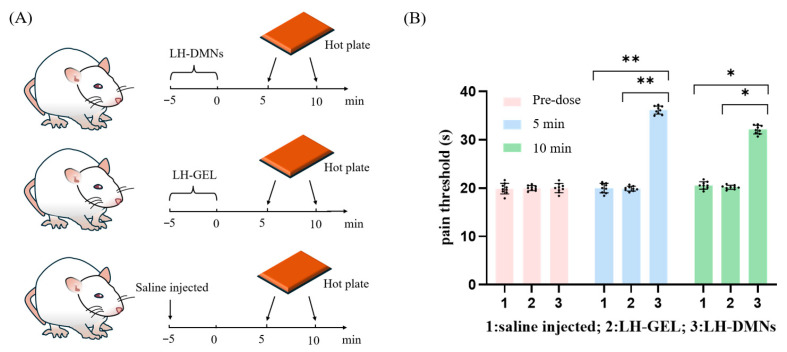
(**A**) Schematic of the hot plate test for analgesia in mice; (**B**) mice hot plate pain thresholds before and at 5/10 min after different analgesia treatments. * *p* < 0.05, ** *p* < 0.01.

We evaluated the local analgesic efficacy of LH-DMNs using the hot plate assay (Figure 4A). Mice exhibiting comparable baseline pain thresholds were randomly divided into three groups: saline control, lidocaine hydrochloride gel (application of LH-GEL), and LH-DMN groups (Figure 4A). Before administration, there was no significant difference in pain threshold among the three groups. At 5 min post-administration, LH-DMNs achieved an 80% elevation in pain threshold (36.11 ± 1.62 s) versus controls (20.00 ± 1.58 s) and LH-GEL (19.89 ± 0.60 s). This superior analgesia persisted at 10 min, with LH-DMNs maintaining a 60% higher threshold (32.00 ± 1.41 s) than both comparators (Table 2). The observed decline in analgesic efficacy of LH-DMNs from 5 min to 10 min reflects the characteristic burst release profile of dissolvable microneedle systems, where rapid polymer dissolution enables immediate drug availability, but limits sustained release. Conventional gels conversely exhibit slower drug permeation through the stratum corneum, leading to delayed onset, yet prolonged action. This pharmacokinetic trade-off between speed and duration is inherent to transdermal delivery platforms. Notably, LH-DMNs maintained statistically superior analgesia versus controls at 10 min, *p* < 0.05, highlighting their clinical utility for procedures requiring rapid anesthesia. In the event of a future requirement to extend the duration of action beyond rapid local anesthesia, combining it with sustained-release polymers could be considered.

The significantly prolonged thermal latency in LH-DMN-treated mice, evidenced by delayed foot-licking responses and sustained threshold increases, demonstrates rapid onset and potent local anesthesia unmatched by conventional topical formulations.

## 4. Discussion

The imperative for rapid and minimally invasive local anesthesia in diverse clinical settings underscores the limitations of conventional approaches. Subcutaneous injections, while effective, are invasive and provoke anxiety and procedural pain [8]. Topical formulations, such as gels and creams, offer non-invasive application, but are fundamentally constrained by the formidable stratum corneum barrier, resulting in delayed onset and unpredictable drug absorption [18,19,20,21]. Microneedle technology has emerged as a promising strategy to bridge this critical gap, facilitating direct intradermal delivery. In this study, LH-DMNs were successfully fabricated to achieve the transdermal delivery of anesthetic drugs.

Through screening and optimization, we selected PVA as the matrix material for the LH-DMNs and developed a two-step fabrication method, yielding a detachable microneedle patch. While both polymers offer biocompatibility and water solubility, the hygroscopic nature of PVP led to unacceptable structural degradation under ambient storage conditions within one month, compromising its practical utility. And the LH-DMNs consistently demonstrated adequate mechanical integrity and provided the requisite mechanical strength of >0.23 N per needle to reliably breach the stratum corneum, confirming their functional efficacy for transdermal drug delivery. HPLC quantification confirmed a drug payload of 24.0 ± 2.84 mg per patch, sufficient for local anesthesia and achieved without compromising microneedle strength, a feature critical for bypassing the stratum corneum to deliver therapeutic LH concentrations. Additionally, in vitro penetration tests showed that the backing layer of the microneedles could be smoothly peeled off after partial dissolution of the needle body, and the microneedles completely dissolved within 3 min, confirming their ability to rapidly release the drug. In vivo hot plate analgesic tests demonstrated that LH-DMNs elevated pain thresholds by 80% versus controls at 5 min post-administration, reaching 36.11 ± 1.62 s, a statistically significant increase over both the control and LH-GEL groups. This immediate analgesic onset confirms successful subcutaneous delivery of therapeutic lidocaine concentrations through rapid stratum corneum penetration.

The success of the LH-DMNs can be attributed to their ability to overcome the primary barrier to transdermal drug delivery—the stratum corneum. By mechanically creating microchannels and subsequently dissolving in situ, the LH-DMNs ensure direct deposition and rapid release of LH into the dermal layers where nociceptors reside. This bypasses the slow passive diffusion required for topical gels, explaining the dramatically accelerated onset of action. These results collectively validate our design rationale: PVA-based DMNs synergize precise physical penetration with rapid payload release to bridge the critical gap between injectable speed and transdermal convenience.

## 5. Conclusions

As a pivotal clinical procedure used in surgical, diagnostic, and chronic pain settings, effective local anesthesia demands delivery systems that reconcile rapid onset with minimal invasiveness [3,4,5]. This study investigated lidocaine hydrochloride-loaded microneedles (LH-DMNs) for local anesthesia, evaluating their drug delivery feasibility, screening for optimal matrix materials, and optimizing a scalable fabrication process. The LH-DMNs demonstrated adequate mechanical strength, rapid dissolution, high biocompatibility, and safety profiles. Compared with traditional transdermal formulations, such as gel and creams, LH-DMNs successfully overcame the stratum corneum barrier, significantly improving transdermal drug absorption efficiency and enabling rapid drug release. This painless, minimally invasive system merges the rapid onset of injections with the sustained delivery of transdermal therapies, offering a promising alternative for clinical analgesia. Further development of LH-DMNs will focus on augmenting drug payloads to prolong analgesic duration, potentially enabling broader clinical applications. And the developed PVA-based microneedle system provides a foundation for future integration of diagnostic functions, such as biomarker detection or closed-loop theranostic systems. With microneedle technology rapidly advancing for diverse therapeutic applications, this platform is poised to revolutionize drug delivery, particularly in precision medicine and personalized therapies.

## Figures and Tables

**Table 1:** Determination of the drug content of lidocaine hydrochloride in microneedles (*n* = 3).

Sample	Sample Solution Peak Area (mV/min)	Sample Solution Concentration (mg/mL)	Dilution Volume (mL)	Content per Sample (mg)
1	1,175,266	0.24235	100	24.235
2	1,025,464	0.21071	100	21.071
3	1,294,303	0.26749	100	26.749

**Table 2:** Effect of lidocaine hydrochloride on pain threshold in mice (s, x ± s, *n* = 9).

Group	Pre-Dose	5 min	10 min
Control	19.78 ± 0.97	20 ± 1.58	20.56 ± 1.13
LH-DMNs	20 ± 1.22	36.11 ± 1.62 **	32 ± 1.41 *
LH-GEL	20 ± 0.5	19.89 ± 0.6	20.22 ± 0.67

* *p* < 0.05, ** *p* < 0.01.

## Data Availability

The raw data supporting the conclusions of this article will be made available by the authors on request.

## Abbreviations

The following abbreviations are used in this manuscript:

LH-DMNs	Lidocaine hydrochloride-loaded dissolvable microneedles
PDMS	Polydimethylsiloxane
PVA	Polyvinyl alcohol
PVP	Polyvinylpyrrolidone
LH-GEL	Lidocaine hydrochloride gel
SEM	Scanning electron microscopy
HPLC	High-performance liquid chromatography
HE	Hematoxylin and eosin
